# Diversity in rhizospheric microbial communities in tea varieties at different locations and tapping potential beneficial microorganisms

**DOI:** 10.3389/fmicb.2022.1027444

**Published:** 2022-11-10

**Authors:** Zheng Zhang, ShiBei Ge, Li-Chao Fan, Shuai Guo, Qiang Hu, Golam Jalal Ahammed, Peng Yan, Li-Ping Zhang, Zheng-Zhen Li, Jian-Yang Zhang, Jianyu Fu, Wenyan Han, Xin Li

**Affiliations:** ^1^Key Laboratory of Tea Quality and Safety Control, Ministry of Agriculture and Rural Affairs, Tea Research Institute, Chinese Academy of Agricultural Sciences, Hangzhou, China; ^2^College of Natural Resources and Environment, Northwest A&F University, Yangling, Shaanxi, China; ^3^Hangzhou Botanical Garden, Hangzhou West Lake Academy of Landscape Science, Hangzhou, China; ^4^College of Horticulture and Plant Protection, Henan University of Science and Technology, Luoyang, China

**Keywords:** microbial communities, soil physicochemical properties, tea varieties, AMF, disease resistance, rhizosphere

## Abstract

Soil microenvironments and plant varieties could largely affect rhizosphere microbial community structure and functions. However, their specific effects on the tea rhizosphere microbial community are yet not clear. Beneficial microorganisms are important groups of microbial communities that hold ecological functionalities by playing critical roles in plant disease resistance, and environmental stress tolerance. *Longjing43* and *Zhongcha108* are two widely planted tea varieties in China. Although *Zhongcha108* shows higher disease resistance than *Longjing43*, the potential role of beneficial tea rhizosphere microbes in disease resistance is largely unknown. In this study, the structure and function of rhizosphere microbial communities of these two tea varieties were compared by using the Illumina MiSeq sequencing (16S rRNA gene and ITS) technologies. Rhizosphere soil was collected from four independent tea gardens distributed at two locations in Hangzhou and Shengzhou cities in eastern China, *Longjing43* and *Zhongcha108* are planted at both locations in separate gardens. Significant differences in soil physicochemical properties as demonstrated by ANOVA and PCA, and distinct rhizosphere microbial communities by multiple-biotech analyses (PCoA, LEfSe, Co-occurrence network analyses) between both locations and tea varieties (*p* < 0.01) were found. Functions of bacteria were annotated by the FAPROTAX database, and a higher abundance of Nitrososphaeraceae relating to soil ecological function was found in rhizosphere soil in Hangzhou. LDA effect size showed that the abundance of arbuscular mycorrhizal fungi (AMF) was higher in *Zhongcha108* than that in *Longjing43*. Field experiments further confirmed that the colonization rate of AMF was higher in *Zhongcha108*. This finding testified that AMF could be the major beneficial tea rhizosphere microbes that potentially function in enhanced disease resistance. Overall, our results confirmed that locations affected the microbial community greater than that of tea varieties, and fungi might be more sensitive to the change in microenvironments. Furthermore, we found several beneficial microorganisms, which are of great significance in improving the ecological environment of tea gardens and the disease resistance of tea plants. These beneficial microbial communities may also help to further reveal the mechanism of disease resistance in tea and potentially be useful for mitigating climate change-associated challenges to tea gardens in the future.

## Introduction

Rhizosphere microbial communities play crucial roles in facilitating plant nutrient uptake, defense and immunity (Aziz et al., 2021; Li J. T. et al., 2021; Chand et al., 2022). As soil microorganisms control the global element cycle in the process of life and death, living soil microorganisms are the main engine of terrestrial biogeochemistry, which promote the turnover of soil materials (Sokol et al., 2022). The roots of most terrestrial plants are occupied by mycorrhizal fungi, which play key roles in regulating soil structure, microenvironments, and ecosystem functions. In recent years, a large number of studies have focused on the structure and functions of plant rhizosphere microbial communities (Dastogeer et al., 2020; Bhantana et al., 2021; Qin et al., 2021; Khaliq et al., 2022). Soil physicochemical properties such as soil type, temperature and humidity, pH, and agronomic measures (e.g., fertilization and pesticide application) affect the composition and diversity of rhizosphere microbial communities (Liu et al., 2021; Liu S. B. et al., 2022). Therefore, determining the complexity and stability of bacteria and fungi in the rhizosphere microbial community and identifying the potential key species have become hot topics (Nwachukwu and Babalola, 2021).

Beneficial microorganisms are important groups of microbial communities that hold ecological functionalities by playing critical roles in plant disease resistance and environmental stress tolerance (Diagne et al., 2020; Weng et al., 2022). Mutualistic symbiosis of arbuscular mycorrhizal fungi (AMF) with host plant roots could provide host plants the essential mineral elements, such as phosphorus (Almario et al., 2022), and facilitate plant health, thus considered as beneficial microbes for the plant (Weng et al., 2022). Besides, beneficial microbes such as Nitrososphaeraceae bacteria could also play crucial roles in ecological function (Shen et al., 2012; Amoo and Babalola, 2017).

Tea (*Camellia sinensis* (L.) O. Ktze.) is a perennial evergreen broad-leaved cash crop, and distributed in areas with high temperature and humidity (Wang et al., 2016). The structure and functions of soil microbes in tea gardens in different environments (such as organic vs. conventional management, geographical location, standing age, different N fertilization, and land use conversion from woodland to tea gardens) and the correlation with soil physical and chemical properties were well studied (Gui et al., 2021; Fan et al., 2022; Gui et al., 2022). At present, tea gardens are facing the problem of soil quality decline and serious diseases (Pandey et al., 2021; San Le et al., 2022). *Longjing43* is one of the most widely planted tea varieties in China. However, it is susceptible to anthracnose, which is one of the most devastating diseases for tea plants, affecting the yield and quality of tea. *Zhongcha108* was generated from *Longjing43* through irradiation-based mutation breeding and it is resistant to anthracnose (Wang et al., 2016). Previous studies revealed that the resistance against anthracnose in *Zhongcha108* is mediated by the hypersensitive response and H_2_O_2_ accumulation (Wang et al., 2018). However, the potential role of beneficial rhizosphere microbes in tea plant resistance to anthracnose is largely unknown.There are some specific microorganisms in tea plants that could induce auxin and iron carriers to resist root rot, for instance, Actinomycetes and Bacillus can promote the resistance of tea plants to leaf blight and tea white scab disease (Wang et al., 2021). Besides, the beneficial rhizosphere microorganisms like AMF can be used to improve soil quality and control tea garden diseases, and nitrogen-fixing bacteria and ammonia-oxidizing bacteria in the rhizosphere can regulate nutrient utilization of tea plants and N cycling of soil (Chen et al., 2011; Bhattacharjee et al., 2012).

Previous studies revealed that rhizosphere microbial communities are susceptible to changes in environmental and plant varieties (Lee et al., 2021; Munoz et al., 2021). However, it is still unclear how tea varieties and soil characteristics affect the structure and functions in rhizosphere bacterial and fungal communities, and the information on beneficial microbial groups is largely lacking. Therefore, identifying the assembly of bacterial and fungal communities under different environmental factors and plant varieties could help us to acknowledge the role of microorganisms in plant management, and further obtain key information about plant growth and health (Babalola et al., 2021). Here, we collected rhizosphere soil from two locations each with two tea varieties with different resistance to pathogens, to analyze the diversity of rhizosphere fungal and bacterial communities by 16S rRNA gene and ITS amplicon sequencing technologies. Our study aims to answer the following research questions:

1. Do tea varieties vs. locations lead to significant changes in tea rhizosphere microbial communities? If yes, which one is more impactful?
2. Do bacteria and fungi respond similarly to the different tea varieties and locations? If not, which one (bacteria or fungi) is more sensitive to the change in microenvironments?
3. What are the beneficial microbial groups in the tea rhizosphere? Are there any potential beneficial microorganisms that could contribute to the higher disease resistance of *Zhongcha108*?

## Materials and methods

### Experimental design and rhizosphere soil sampling

Sampling was conducted from two locations with different soil nutrient conditions in Zhejiang, China: tea gardens of the Tea Research Institute of the Chinese Academy of Agricultural Sciences, Hangzhou (120°09′ E, 30°14′ N) and the tea gardens of the Shengzhou integrated experimental base, Shengzhou (120°48′ E, 29°75′ N). Two tea varieties (Longjing43, and Zhongcha108) were being cultivated in both sampling sites since 2013 and were managed under similar management practices and fertilization routines. Briefly, there were four tea gardens in this study, two of them were located in Hangzhou City and the rest two were located in Shengzhou City. In the same location, both two tea varieties were planted in different tea gardens. Urea (450 kg·hm-2) was used as germination-accelerating fertilizer in the middle of February, and compound fertilizers (750 kg· hm-2; N:P2O5:K2O, 15: 15: 15) were applied before the heavy pruning of spring tea in May, and rapeseed cake (4,500 kg·hm-2) was used as base fertilizer in the first 10 days of October every year. Tea plants were light pruned after the spring tea harvesting, the second pruning was done in mid-July, and the third topping pruning was performed at the end of November each year.

In each tea garden, we randomly selected eight sampling plots, and in each plot (6*6 m2)，five tea plants were randomly selected for rhizosphere soil sampling. The rhizosphere soil of each tea garden was mixed into three samples for the analysis of soil physicochemical properties. All samples were collected and stored in the ice box, then brought them back to the laboratory and stored at −80°C for subsequent DNA extraction in 4 h. The rhizosphere soils of Longjing43 and Zhongcha108 in Hangzhou were recorded as HZ-LJ43 and HZ-ZC108, respectively. The rhizosphere soils of Longjing43 and Zhongcha108 in Shengzhou were recorded as SZ-LJ43 and SZ-ZC108, respectively.

### Soil physico-chemical properties analysis

Soil pH was determined by a combination of glass electrodes using a 1: 2.5 (w: v) ratio of soil to distilled water (Mi et al., 2018). Soil total C (TOC) and N (TON) were measured by LECO CNS Combustion Analyzer (LECO, CNS 2000, LECO Corporation, Michigan, United States) following manufacturer protocol. Available phosphorus (AP) in soil was determined by ammonium fluoride extraction (Bray and Kurtz, 1945). Soil available K^+^, Ca^2+^, and Mg^2+^ were extracted using 1 M KCl (1:10), and determined by using atomic absorption spectrophotometry (NovAA300, Analytik Jena, Germany).

### Detection and photographing of AMF colonization rate

The AMF used in the experiment is *Rhizophagus irregularis* (syn. *Globus intraradices*). Corn plants were used to propagate fungal spores. After 3 months of low phosphorus cultivation, corn roots were cut up, mixed with sand, and stored at 20°C for later use. Sixhundred spores/pot (25 g) were added to potted tea varieties *Longjing43* and *Zhongcha108*. After 60 days, the root length colonization rate (RLC%) was detected.

The RLC% of tea roots was detected by trypan blue staining (Phillips and Hayman, 1970). The roots of plants inoculated with AMF spores for 60 days were cleaned, cut into about 1 cm root segments, immersed in 10% KOH (w/v), and bathed at 95°C for 30–40 min. After cooling and cleaning, root samples were immersed in 2% HCl and acidified for 5 min. After removing the liquid, 0.05% trypan blue reagent was added (trypan blue dissolved in lactic acid glycerin reagent, w/v), and placed in a water bath at 95°C for 10 min, afterward, the reagent was removed after cooling, and lactic acid glycerin reagent (lactic acid: glycerin: water =8:1:1) was used to decolorize root pigments at room temperature for more than 24 h. Randomly selected root segments were placed on a grid-line slide, observed under a microscope (Leica Microsystems, Germany) at 20x magnification, and counted according to the grid-line inter-sect method (Giovannetti and Mosse, 1980). Five replicates were counted for each garden.

### Rhizosphere soil microbial DNA extraction and PCR amplification

SDS cracking fluid freezing and thawing method was used for DNA extraction, genomic DNA extraction was accomplished by PowerMax extraction kit (MoBio Laboratories, Carlsbad, CA, United States), and thereafter the DNA gel was stored at −20°C. DNA quantity and quality were determined by using NanoDrop ND-1000 luminance meter (Thermo Fisher Scientific, Waltham, MA, United States). The primer sequences of bacteria and fungi are two sets: 1) 16S: forward primers 515F (5′-GTGCCAGCMGCCGCGGTAA-3′) and reverse primers 806R (5′-GGACTACHVGGGTWTCTAAT-3′); 2) ITS: forward primers ITS1 (5’-CTTGGTCATTTAGagGaAGTAA-3′) and reverse primers ITS2 (5 ‘-GCTGCGTTCTTCATCGATGC-3′). Barcode was synthesized into the sequence by using a specific 7-BP specific sequence. Phusion high-fidelity PCR Master Mix with HF Buffer was used for the PCR reaction system 50 μl: 25 μl. Three microliter (10 μM) F/R primers, 10 μl DNA sample, 6 μl ddH_2_O. The PCR system was amplified according to the following reaction conditions: pre-denaturation 98°C for 30s, followed by 25 cycles: denaturation 98°C for 15 s annealing 58°C for 15 s extension 72°C for 15 s. The final extension is 72°C for 1 min. PCR products were purified with AMPure XP Beads (Beckman Coulter, Indianapolis, IN). Quantification was performed by using PicoGreen dsDNA Assay Kit (Invitrogen, Carlsbad, CA, United States). After quantification, Illumina Novaseq 6,000 pin-end 2 × 150 bp platform was used for further sequencing.

### Sequence analysis

The Quantitative Insights Into Microbial Ecology (QIIME, v1.9.0) pipeline was employed to process the sequencing data, as previously described (Caporaso et al., 2010). Briefly, raw sequencing reads with exact matches to the barcodes were assigned to respective samples and identified as valid sequences. The low-quality sequences were filtered through the following criteria (Gill et al., 2006; Chen and Jiang, 2014): sequences that had a length of <150 bp, had average Phred scores of <20, contained ambiguous bases, and contained mononucleotide repeats of >8 bp. Paired-end reads were assembled by using Vsearch V2.4.4 (−-fastq_mergepairs--fastq_minovlen 5), operational taxonomic unit (OTU) was picked by using Vsearch V2.4.4,included Dereplication (−-derep_fulllength), cluster (−-cluster_fast,--id 0.97), detection of chimeras (−-uchime_ref) (Rognes et al., 2016). A representative sequence was selected from each OTU by using default parameters. OTU taxonomic classification was conducted by VSEARCH searching the representative sequences set against the Greengene database.

The OTU table was further generated to record the abundance of each OTU in each sample and the taxonomy of these OTUs. OTUs containing less than 0.001% of total sequences across all samples were discarded. To minimize the difference in sequencing depth across samples, an average, rounded rarefied OTU table was generated by averaging 100 evenly resampled OTU subsets under 90% of the minimum sequencing depth for further analysis.

### Bioinformatics and statistical analysis

Sequence data analyses were mainly performed by using QIIME and R (v3.2.0) packages. OTU-level alpha diversity indices (Chao1 richness estimator, Shannon diversity index, and Simpson index) were calculated. OTU-level ranked abundance curves were generated to compare the richness and evenness of OTUs among samples.

Beta diversity was analyzed to investigate the structural variation of microbial communities between samples by using UniFrac distance metrics (Lozupone et al., 2007) and visualized *via* principal coordinate analysis (PCoA) (Ramette, 2007). Differences in the Unifrac distances for pairwise comparisons among groups were determined using Student’s t-test and the Monte Carlo permutation test with 1,000 permutations. Venn diagram was generated to visualize the shared and unique OTUs among samples or groups using the R package “VennDiagram,” based on the occurrence of OTUs across samples/groups regardless of their relative abundance (Zaura et al., 2009). Taxa abundances at the phylum levels were statistically compared among samples or groups by Kruskal.test from the R stats package. The co-occurrence patterns were constructed by calculating multiple correlations and similarities within the network, Pairwise Pearson correlations were calculated between the remaining OTUs. A valid co-occurrence was considered as a statistically robust correlation between taxa when Pearson’s correlation (r) was >0.7 and the *value of p* was <0.01. Each node indicated an individual OTU, and each edge represented the pairwise correlations between nodes standing for a significant metabolic association in the network. The co-occurrence was calculated and visualized using igraph package. We generated 1,000 Erd˝os–R’enyi random networks with each edge having the same probability of being assigned to any node to compare with the topology of the real network. All statistical analyses were performed using R (v4.0.2) unless otherwise stated.LEfSe (Linear discriminant analysis effect size) was performed to detect differentially abundant taxa across groups using the default parameters (Segata et al., 2011). FAPROTAX is a database that maps prokaryotic clades (e.g., genera or species) to established metabolic or other ecologically relevant functions (Louca and Doebeli, 2016), the significant difference bacterial function was annotated, and spearman rank correlation was used to analyze the correlation between different bacterial species, ecological functions and metabolic pathways.

The experimental data (soil physical and chemical properties, AMF colonization rate and relative abundance of microorganisms) were statistically analyzed by SPSS19.0 and figures were drawn by R and GraphPad prism8.

## Results

### Soil physicochemical properties

As rhizosphere microbial communities are susceptible to the microenvironments, we firstly explored the soil physico-chemical properties, including soil pH, the content of soil total C (TOC) and N (TON), available phosphorus (AP), soil available K^+^ (AK), Ca^2+^ (ACa), and Mg^2+^ (AMg), of different tea varieties in different locations. Results revealed that soil pH, and the contents of AK, ACa and AMg in Shengzhou gardens were significantly higher than that in Hangzhou (*p* < 0.01). There is no significant difference in the contents of TOC and TON between the tea gardens in Shengzhou and Hangzhou. Even the TOC and TON contents were much higher in *Longjing 43*, than that in *Zhongcha108* in Hangzhou, however, there is no difference between those two varieties in the Shengzhou location. The contents of AP in Hangzhou were significantly higher than that in Shengzhou gardens (*p* < 0.01; Table 1).

**Table 1:** Contents of some major soil nutrients and soil pH in different tea gardens with different tea varieties.

Soil type	HZ-LJ43	HZ-ZC108	SZ-LJ43	SZ-ZC108	*F*-value	*P*-value
TOC(%)	3.55 ± 0.31a	1.61 ± 0.06c	1.96 ± 0.06b	1.84 ± 0.12bc	81.09	2.5E-06
TON(%)	0.3 ± 0.02a	0.14 ± 0.01b	0.16 ± 0.01b	0.16 ± 0.01b	80.77	2.53E-06
pH	3.86 ± 0.01d	4.34 ± 0.02c	4.57 ± 0.05a	4.48 ± 0.02b	334.86	9.56E-09
AP (mg/kg)	262.56 ± 12.13b	126.93 ± 6.34c	27.43 ± 1.22a	25.5 ± 2.52a	769.19	3.5E-10
AK (mg/kg)	179.18 ± 14.59a	173.45 ± 10.31a	306.52 ± 2.93b	288.91 ± 18.97b	86.75	1.92E-06
ACa (mg/kg)	222.7 ± 6.6d	358.93 ± 27.9c	538.16 ± 13.10a	406.14 ± 4.6b	200.6	7.25E-08
AMg (mg/kg)	26.82 ± 1.06d	34.3 ± 1.37c	88.81 ± 0.81a	67.24 ± 0.53b	2567.09	2.85E-12

Mean values (*n* = 3) ± STDEV followed by different letters indicate a significant difference (*p* < 0.05, LSD’s test). TOC, TON (%), AP, AK, Aca, AMg (mg/kg).

### Rhizosphere microbial community structure

**Figure 1:**
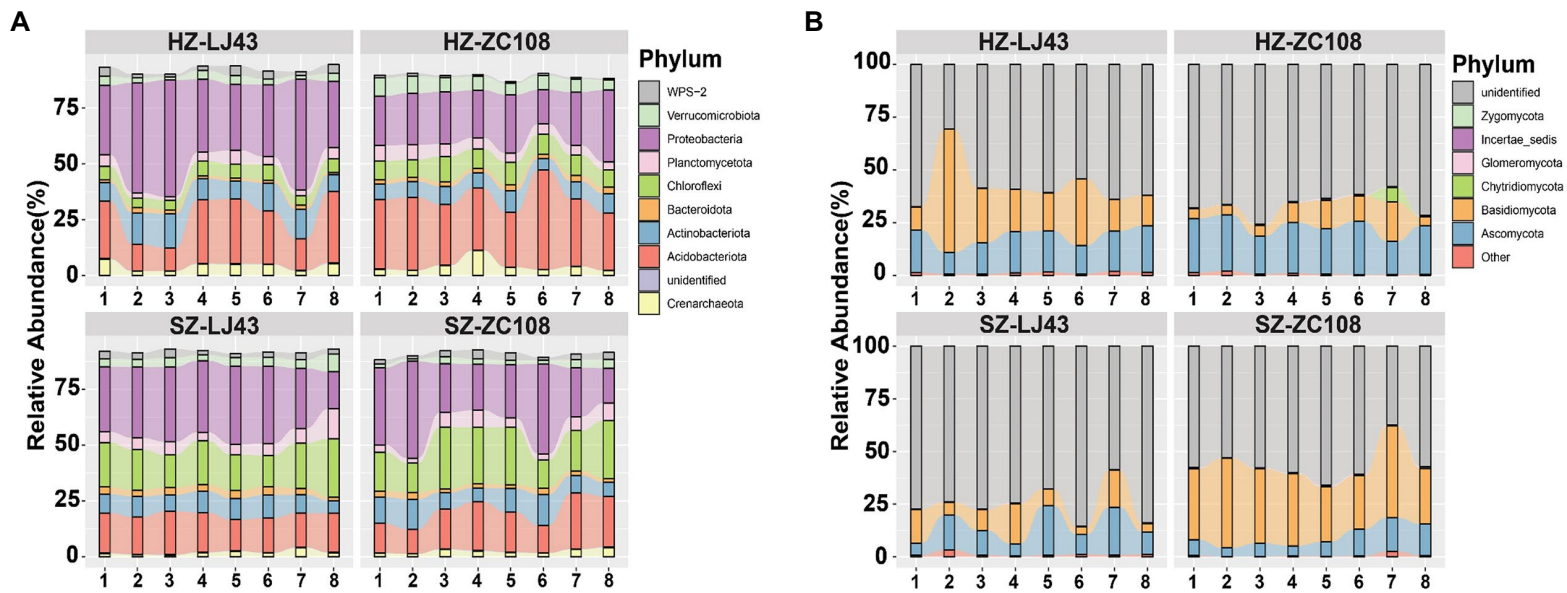
The composition of rhizosphere microbial communities of two tea varieties in different locations. Phylum distribution of the OTUs. **(A)** Top 10 bacterial phyla abundances in rhizosphere soil of HZ-LJ43, HZ-ZC108, SZ-LJ43, and SZ-ZC108. **(B)** Top 10 fungal phyla abundances in rhizosphere soil of HZ-LJ43, HZ-ZC108, SZ-LJ43, and SZ-ZC108. Mean values ± standard error (*n* = 8); different letters indicate a significant difference (*p* < 0.05) based on one-way ANOVA followed by an LSD test.

To assess the rhizosphere microbial community structure, we compared the composition and abundance of rhizosphere microorganisms in tea plants and the co-occurrence of microbial communities in different locations and varieties. Sequences were clustered into Operational taxonomic units (OTU) with a 97% threshold, and diluted to the same sequencing depth. These sequences were divided into nine dominant bacteria phyla, including Proteobacteria(38%), Crenarchaeota (4%), Acidobacteriota (22%), Actinobacteriota (11%) (Figure 1A), and five dominant fungal phyla, such as Ascomycota (17%), Basidiomycota (24%), and unidentified (57%) (Figure 1B).

We compared the differences in bacterial phyla between the four gardens, and found that Crenarchaeota was the phylum with higher relative abundance in Hangzhou, the relative abundance of Crenarchaeota in HZ-LJ43 (4.1%) and HZ-ZC108 (4.1%) were higher than that in SZ-LJ43 (1.9%) and SZ-ZC108 (2.5%). The relative abundance of Acidobacteriota in HZ-LJ43 (21.9%) and HZ-ZC108 (30.5%) were higher than SZ-LJ43 (16.8%) and SZ-ZC108 (17.7%); The relative abundance of Chloroflexi was higher in Shengzhou (SZ-LJ43, 18.6% and SZ-ZC108, 20.8%) than that in Hangzhou (HZ-LJ43, 5.6% and HZ-ZC108, 9%) (*p* < 0.01; Figure 1A; Supplementary Table S1).

While comparing the differences in bacterial phyla between the two varieties, it was found that Proteobacteria was the phylum with higher relative abundance in LJ43, and the relative abundance of Proteobacteria in HZ-LJ43 (38.3%) and SZ-LJ43 (30%) was higher than that in HZ-ZC108 (23.4%) and SZ-ZC108 (27.7%). However, there was no bacterial phylum with significantly higher relative abundance in *Zhongcha108* (*p* < 0.01; Figure 1A; Supplementary Table S1).

Next, we compared the differences in fungal phyla between the four gardens, and found that Ascomycota was the phylum with higher relative abundance in Hangzhou gardens, the relative abundance of Ascomycota in HZ-LJ43 (17.4%) and HZ-ZC108 (22.5%) were higher than SZ-LJ43 (13.2%) and SZ-ZC108 (9.1%); the relative abundance of Zygomycota in HZ-LJ43 (0.05%) and HZ-ZC108 (0.1%) were higher than that in SZ-LJ43 (0.03%) and SZ-ZC108 (0.01%) (*p* < 0.01; Figure 1B; Supplementary Table S2). However, there is no fungal phylum with a significantly higher relative abundance in Shengzhou gardens than that in Hangzhou gardens. While comparing the differences in fungal phyla between the two varieties, it was found that Glomeromycota was the phylum with higher relative abundance in HZ-ZC108 (0.36%) and SZ-ZC108 (0.54%) than that in HZ-LJ43 (0.01%) and SZ-LJ43 (0.1%). We did not find the fungal phylum with higher relative abundance in LJ43 than that in ZC108 (*p* < 0.01; Figure 1B; Supplementary Table S2).

It is revealed that bacterial phyla such as Crenarchaeota, Acidobacteriota and Chloroflexi, and fungal phyla such as Ascomycota and Zygomycota, had relative abundance differences between the two locations. Moreover, there were relative abundance differences between the two varieties, including bacterial phylum Proteobacteria and fungal phylum Glomeromycota.

**Figure 2:**
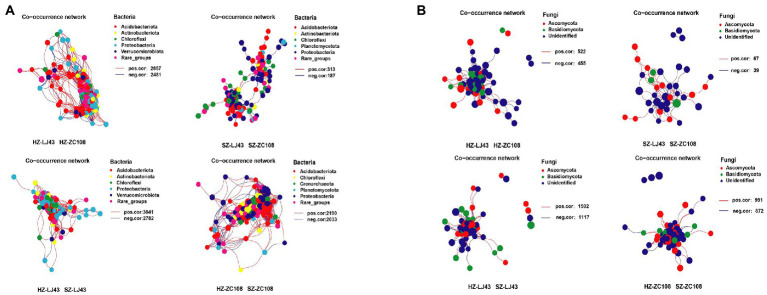
**(A)** The bacterial co-occurrence networks based on OTU correlation analysis. The OTU with a relative abundance above 0.1% was selected. A connection stand for a strong (Pearson’s *r* > 0.7) and significant (*p* < 0.01) correlation. The size of each node is proportional to the relative abundance of each OTU. The red edge represents a positive correlation, and the blue edge represents a negative correlation. **(B)** The fungal co-occurrence networks based on OTU correlation analysis, and the analysis method is the same as **(A)**.

The Co-occurrence edge of microbial communities in different locations was more than that of different varieties of microbes, which indicated that the interactions between microbes in different locations were closer and more complex (Figure 2A). The number of interacting edges between LJ43 and ZC108 bacterial communities in different locations was 5,823 and 4,223, and the number of interacting edges between different bacterial communities in Hangzhou and Shengzhou locations was 5,338 and 500. The connectance of rhizosphere bacterial communities of two varieties in Hangzhou is 40.9, and 5.40% in Shengzhou. The connectance of rhizosphere bacterial communities of two locations in LJ43 is 43.6, and 33.2% in ZC108 (Supplementary Table S3).

The number of interacting edges between the fungal communities of LJ43 and ZC108 in different locations was 2,619 and 1,863, and the number of interacting edges between the fungal communities of different varieties in Hangzhou and Shengzhou was 977 and 106, respectively. The connectance of rhizosphere fungal communities of two varieties in Hangzhou is 28.7%, and that in Shengzhou is 14.3%. The connectance of rhizosphere bacterial communities of two locations in LJ43 is 47.1%, and that in ZC108 is 43.5% (Supplementary Table S4).

The co-occurrence network of tea rhizosphere communities between different locations is more complex than that between different varieties and the interactions between bacterial communities were closer and more complicated than that of fungal communities (*p* < 0.01; Figure 2B; Supplementary Tables S3, S4).

### Alpha diversity of rhizosphere microbial communities

**Figure 3:**
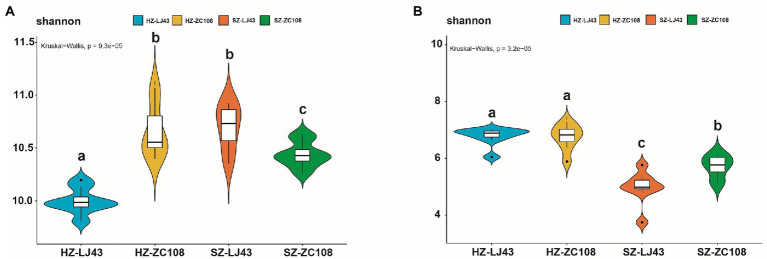
Alpha diversity (α-diversity) estimated as Shannon index. **(A)** Bacteria, **(B)** Fungi.The horizontal lines represent the median. The tops and bottoms of boxes represent 75th and 25th quartiles, respectively. The upper and lower whiskers represent a 95% confidence interval. Mean values ± standard error (*n* = 8); Different letters indicate a significant difference (*p* < 0.05) based on one-way ANOVA followed by an LSD test.

To better prove the influence of location and variety on the tea rhizosphere microbial community, we analyzed the alpha diversity of the microbial community. The alpha diversity of rhizosphere microbial communities, which represents the diversity and abundance of microbial communities, showed significant differences among different tea varieties and locations. Specifically, for the alpha diversity of bacteria, the Shannon index of HZ-ZC108 and SZ-LJ43 was the highest, SZ-ZC108 was the second, and HZ-LJ43 was the lowest (*p* < 0.01) (Figure 3A; Supplementary Table S5). For the alpha diversity of fungi, the Shannon index of Hangzhou was significantly higher than that of Shengzhou, and the Shannon index of *Zhongcha108* was significantly higher than that of *Longjing43* (*p* < 0.01; Figure 3B; Supplementary Table S5). It is found that the microbial abundance in the tea rhizosphere was affected by the location and variety.

### Specific OTUs and β diversity in rhizosphere microorganisms

**Figure 4:**
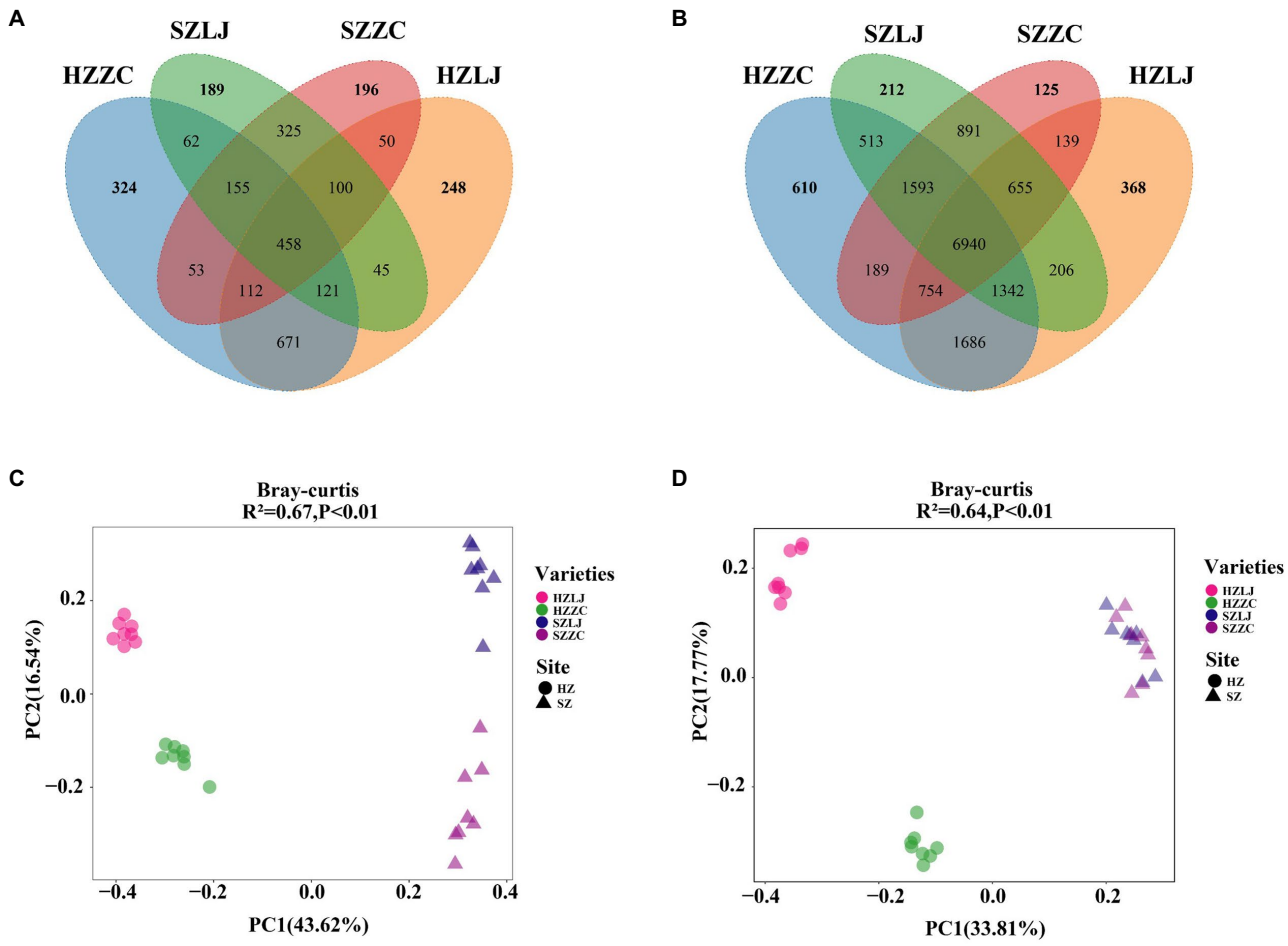
Differences in rhizosphere microbial communities of two tea varieties in different locations. **(A)** The rhizosphere bacteria in HZ-LJ43, HZ-ZC108, SZ-LJ43 and SZ-ZC108 shared OTUs and unique OTUs. **(B)** The rhizosphere bacteria in HZ-LJ43, HZ-ZC108, SZ-LJ43 and SZ-ZC108 shared OTUs and unique OTUs. **(C,D)** Principal Coordinates Analysis (PCoA) using the unweighted Unifrac distance metric. The values of PC1 and PC2 are the percentages that can be explained by the corresponding axis.

To find out the influence of location and variety on the tea rhizosphere microbial community, we analyzed the composition and structure of the microbial community. There were 6,940 identical OTUs in bacterial communities. The unique OTU of each bacterial community was HZ-LJ43 (368), HZ-ZC108 (610), SZ-LJ43 (212), SZ-ZC108 (125). There were 458 identical OTUs in fungal communities. The unique OTU of each fungal community was HZ-LJ43 (248), HZ-ZC108 (324), SZ-LJ43 (189), SZ-ZC108 (125). The number of specific OTU was higher in Hangzhou than that in Shengzhou, and the number of specific Fungal OTU was higher in *Zhongcha108* than that in *Longjing43* (Figure 4A).

The community structure of fungi was affected by both location and variety. Using the bray-Curtis distance to calculate the β diversity between samples, PC 1 and PC 2 explained 43.62 and 16.54% of the differences, respectively. PCoA analysis showed significant differences in the rhizosphere fungal community structure between *Longjing43* and *Zhongcha108*, and the influence of different locations on the fungal community was greater than the influence of variety (R^2^ = 0.67, *p* < 0.01; Figure 4C).

The β diversity of rhizosphere bacteria also was calculated by bray-Curtis distance. PCoA analysis showed that PC1 and PC2 accounted for 33.8 and 17.8% differences, respectively. PCoA demonstrated significant differences in bacterial community structure between HZ-LJ43 and HZ-ZC108, but the bacterial community structure of SZ-LJ43 and SZ-ZC108 were similar (R^2^ = 0.64, *p* < 0.01; Figure 4D). Those results revealed that the microbial community structure in tea rhizosphere was affected by the location and variety.

### LEfSe analysis of rhizosphere fungal community

**Figure 5:**
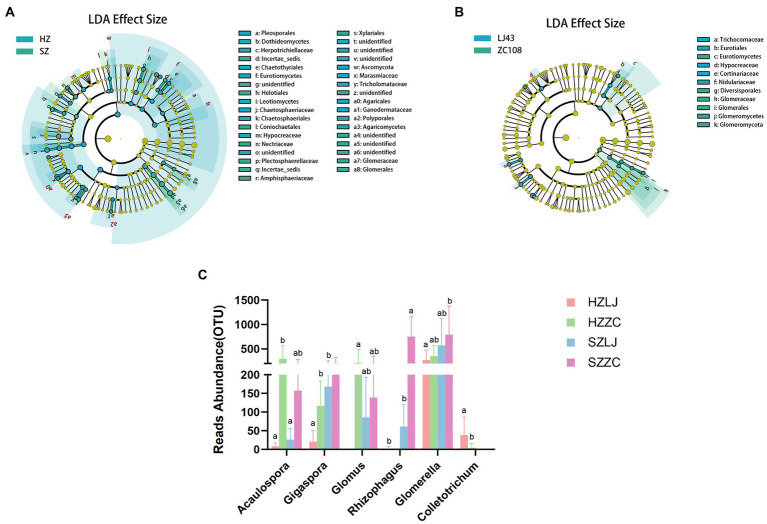
**(A)** LEfSe results for the different fungi in two locations, blue represents the differential fungi in Hangzhou, and green represents the differential fungi in Shengzhou (*p* < 0.05, LDA>2.0). **(B)** LEfSe results for the different fungi in two varieties, blue represents the differential fungi in LJ43, and green represents the differential fungi in ZC108 (*p* < 0.05, LDA>2.0). **(C)** The OTU abundance of AMF and *Colletotrichum*. a and b represent significance at *p* < 0.05.

To screen the rhizosphere fungi affected by location and variety respectively, we conducted LEfSe analysis. LEfSe analysis was based on Linear discriminant analysis (LDA) to screen biomarkers (species with significant differences between groups) among samples. The results showed that compared with *Longjing43*, the Glomerales, Glomeromycetes, Glomeromycota were the dominant biomarker fungi in *Zhongcha108* (*p* < 0.05, LDA > 2.0; Figure 5B). Compared with the fungal differences between the two tea varieties, there were more fungal differences between the two locations (Figure 5A).

According to the results of LEfSe analysis, we analyzed the fungi abundance of ITS amplicon sequencing. We found that the relative abundance of Acaulospora, Gigaspora, Glomus, and Rhizophagus in AMF in *Zhongcha108* was significantly higher than that in *Longjing43* (*p* < 0.01). The relative abundance of Glomerella and Colletotrichum was also significantly different among locations and varieties (*p* < 0.05). Especially, the relative abundance of Colletotrichum in *Longjing43* was significantly higher than that of *Zhongcha108* (*p* < 0.05; Figure 5C; Supplementary Table S6).

Compared with the influence of varieties, we screened out more fungi with different abundance under the influence of location. And we discovered that AMF might be a fungal group affected by the variety.

### FAPROTAX predicted the ecological function of the rhizosphere bacterial community

**Figure 6:**
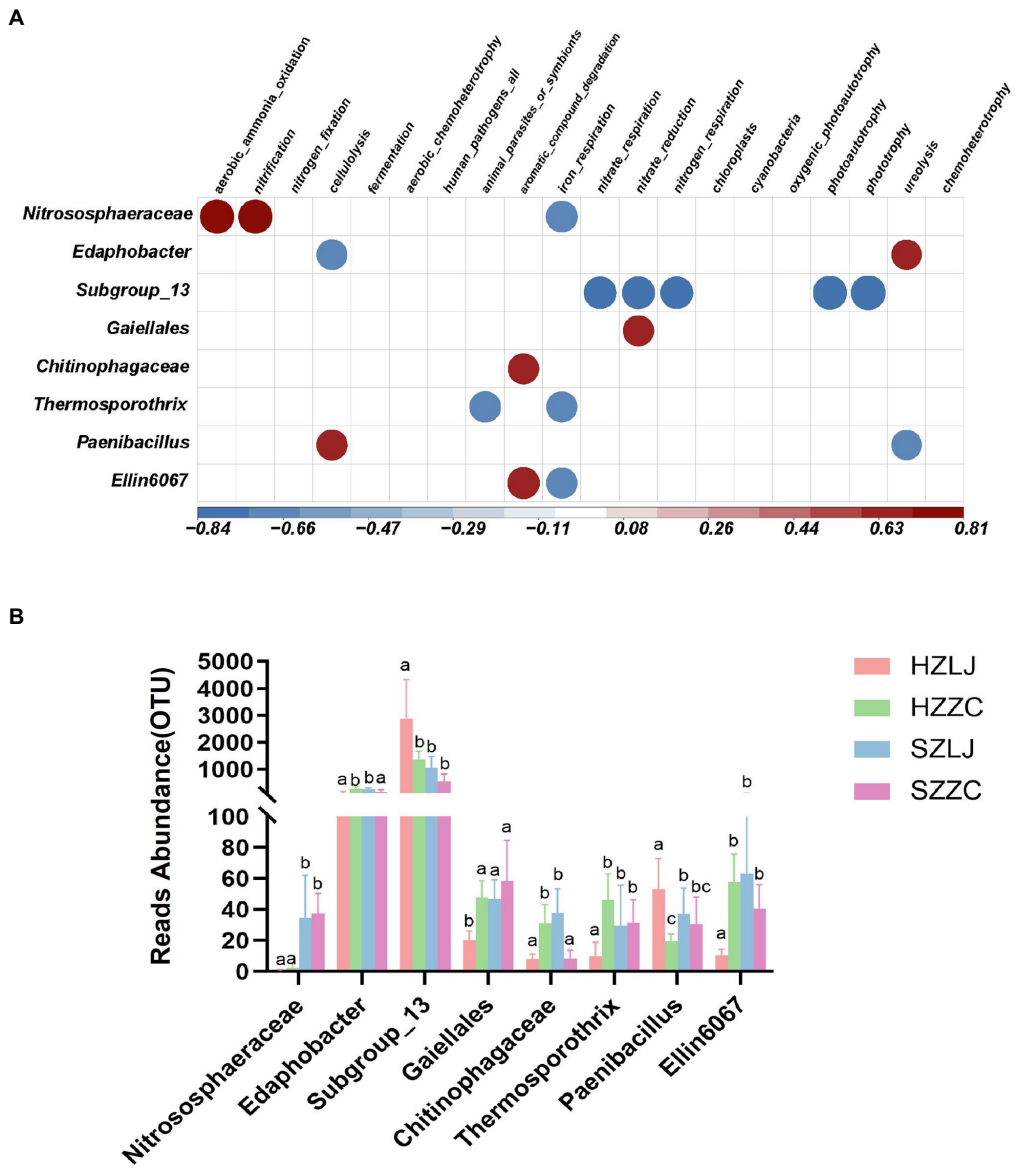
**(A)** Correlation heat map between bacterial OTUs and bacterial ecological function, Red was positively correlated, and blue was negatively correlated (*p* < 0.05). **(B)** The OTU abundance of related bacteria in **(A)**. a, b and c represent significance at *p* < 0.05.

To annotate the function of the tea rhizosphere bacterial community and analyze their influence by location and variety respectively, we annotated the ecological function of bacteria through FAPROTAX database, and analyzed some bacteria significantly related to ecological functions. Based on FAPROTAX, Nitrososphaeraceae was positively correlated with ammonia oxidation and nitrification but negatively correlated with iron respiration function (*p* < 0.05). Subgroup_13 was negatively correlated with nitrate respiration, nitrate reduction, nitrogen respiration, photoautotrophy-related functions and photonutrition (*p* < 0.01). Gaiellales was positively correlated with nitrate reduction (*p* < 0.05). Chitinophagaceae was positively correlated with the degradation of aromatic compounds (*p* < 0.05). Paenibacillus was negatively correlated with urea degradation. Edaphobacter bacteria was positively correlated with urea degradation (*p* < 0.05; Figure 6A). According to the results of FAPROTAX annotation, we analyzed the bacterial abundance of 16 s amplicon sequencing. The relative abundances of Nitrososphaeraceae, Gaiellales, and Ellin6067 were significantly higher in Shengzhou than that in Hangzhou (*p* < 0.05). The relative abundance of Paenibacillus was significantly higher in *longjing43* than that in *zhongcha108* (*p* < 0.05). The relative abundance of Subgroup_13 was significantly higher in Hangzhou than that in Shengzhou, and was significantly higher in *longjing43* than that in *zhongcha108* (*p* < 0.05; Figure 6B; Supplementary Table S7).

### Mycorrhizal staining and colonization rate of two tea varieties

**Figure 7:**
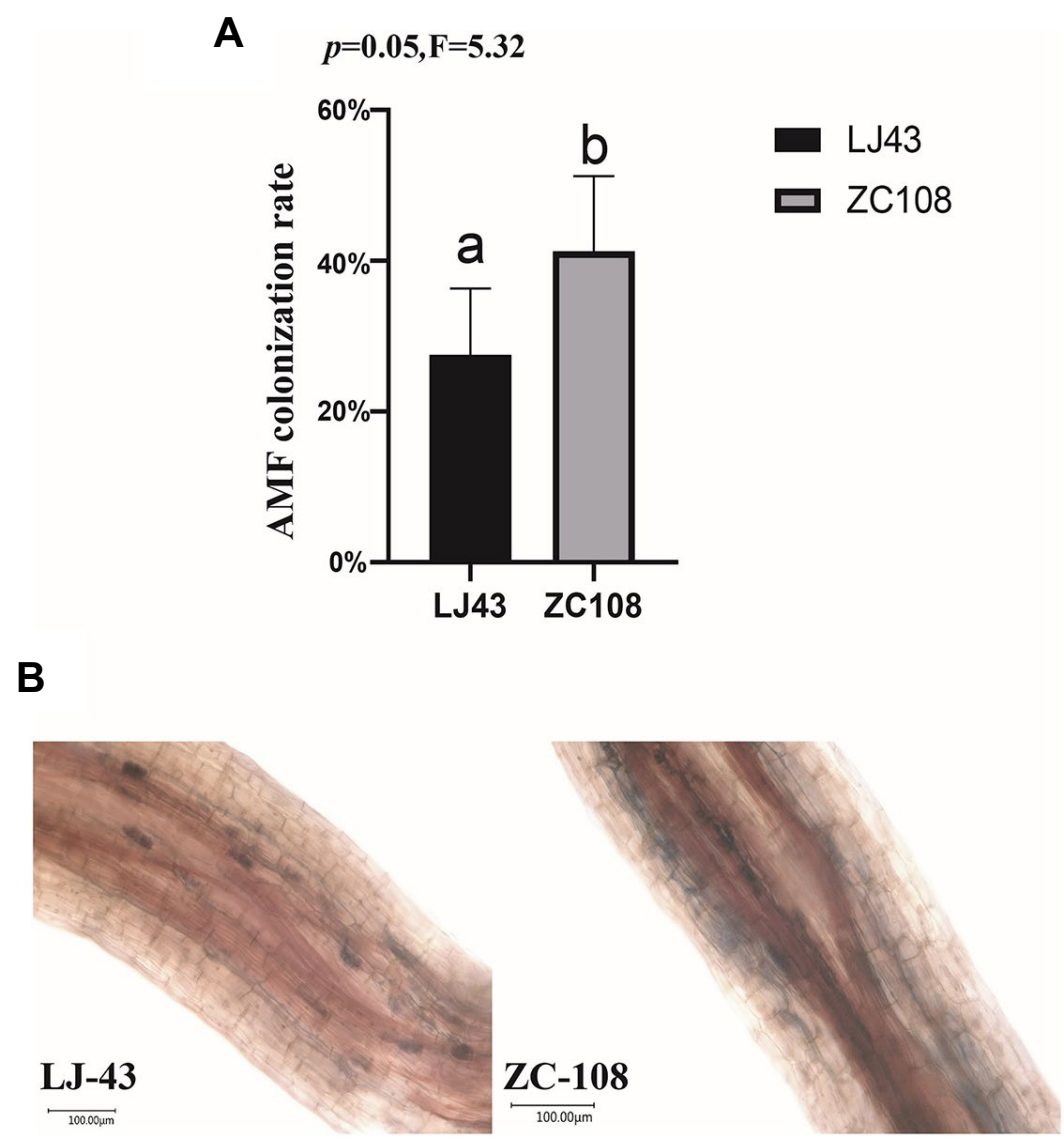
**(A)** Arbuscular mycorrhizal fungi (AMF) colonization rate. The root length colonization rate (RLC%) of two varieties was counted, and 5 replicates were used for each variety. **(B)** Trypan blue stained roots showing AMF colonization in two varieties. Photographs were taken with an Ultra-depth microscope. Mean values ± standard error (*n* = 5); different letters indicate a significant difference (*p* = 0.05) based on one-way ANOVA followed by an LSD test.

To confirm the colonization rate of two tea varieties with AMF, we colonized AMF in the rhizosphere of *Longjing43* and *Zhongcha108* in a field experiment. After 3 months, we stained the roots and determined the colonization rate. It is found that the AMF colonization rate of *Zhongcha108* (41.25%) was higher than that of *Longjing43* (27.54%) (*p* = 0.05; Figure 7A). Trypan blue stained roots of *Longjing43* and *Zhongcha108* as shown in (Figure 7B) were consistent with the rate of AMF colonization on tea plant roots.

## Discussion

The rhizosphere microbial community is an indispensable part of the tea garden ecosystem, which plays an important role in the growth and development of tea plants and the resistance of tea plants to diseases and stresses (Bag et al., 2022). To investigate the effects of variety and environmental factors (such as regional and soil physical and chemical properties) on the rhizosphere microbial communities, and to make better use of beneficial microorganisms, we investigated the rhizosphere microbial communities of two tea locations and two varieties with different disease resistance in Zhejiang Province, China.

This study showed that among the four tea gardens, the pH of the Hangzhou tea garden was lower than that of the Shengzhou tea garden, which may be due to the greater age of the tea garden, resulting in the decrease in the pH of the tea garden soil (Table 1). Previous literature revealed that the bacterial community was significantly negatively correlated with soil pH (Zhou et al., 2017), and it is further confirmed that pH is a key factor affecting the microbial community in tea gardens (Muneer et al., 2022). As shown in Table 1, although the pH values of the four tea gardens are similar, the significance analysis by SPSS shows that the pH values of tea gardens are significantly different among tea gardens (*p* < 0.01), and the microbial abundance of the two locations is also significantly different (Figure 3) (*p* < 0.05). We found that the rhizosphere microorganisms of tea plants were affected at different pH values, for example, the abundance of microorganisms such as Acidobacteriota and Ascomycota was higher in the tea gardens with lower pH at Hangzhou. It is consistent with the results from previous studies, which have also confirmed that these microorganisms are more abundant in acidic soils (Liu H. X. et al., 2022; Liu, J. L. et al., 2022). As tea is an acid-loving plant, it is found that the bacterial community and fungal community in the acid soil of tea gardens are mainly controlled by pH value (Yang et al., 2021). This study has also confirmed that the abundance of rhizosphere fungi in tea garden soils with different pH values is significantly different (Figure 3B), and the abundance of bacterial community is also significantly different, which confirms that pH may be the main factor affecting the microbial community in tea garden rhizosphere (Figure 3A). The content of mineral elements is also an important driving factor affecting the microbial community. Former researchers analyzed the physical and chemical factors affecting the microbial community in China’s oil fields and found that total nitrogen, total phosphorus, available P and other environmental factors in soil were positively correlated with the abundance of the microbial community (Zhang et al., 2010; Macik et al., 2022). C, N, and P in soil significantly affected the diversity of AMF in the rhizosphere (Zhao et al., 2020). In particular, the application of P fertilizer in the soil would significantly improve the diversity of fungi in the soil and rhizosphere, which has also been confirmed in our study. Higher P in tea gardens in Hangzhou may lead to higher fungal abundance (Table 1; Figures 3A,B). Therefore, the location affects the rhizosphere microbial community of tea gardens, and it may be due to the difference in soil physical and chemical properties of tea gardens in different locations (Zhang et al., 2020).

Our study showed that there were differences in the structure of tea rhizosphere microorganisms among different varieties (Figures 1A,B), which was consistent with previous studies on different sugarcane varieties (Nong et al., 2020). Previous studies revealed that there are significant differences in bacterial community structure among maize varieties (Kong et al., 2020), and the structure of the rhizosphere bacterial community was different among different olive varieties (Argyri et al., 2020; Kong et al., 2020). Therefore, it is proposed that variety is the key factor affecting the rhizosphere microbial community (Luo et al., 2021). PCoA analysis showed that locations and varieties had significant effects on the diversity of rhizosphere microorganisms (*p* < 0.05; Figures 3A,B), and this result was also confirmed by venn diagram at otu level (Figures 4A,B). Previous studies have found that there are differences in the rhizosphere bacterial diversity of tea plants in different locations, which is consistent with our findings, but more importantly, we found that varieties are also the main factor affecting the rhizosphere microbial community. As microbial communities are separated by different locations on the PC1 axis, bacterial and fungal communities are still mainly affected by environmental factors (Lopes et al., 2021). Co-occurrence network analysis showed that locations and varieties had significant effects on the interactions of microorganisms.The relationship between microbial communities in different locations is far more complicated than that among different varieties (Figures 2A,B).

Our study confirmed that both varieties and locations can significantly cause changes in rhizosphere bacteria and fungi communities. Finally, we inferred that the location is more impactful than variety in influencing the microbial community, which positively answered the first question. Our results also show that microbial communities respond differently to the different tea varieties and locations, which positively answered the second question. We speculate that fungal communities are more susceptible to locations than bacterial communities because the structure of fungal communities in different locations showed greater differences than that of bacterial communities (PC1:43.62%,PC2:16.54%) (Figures 4C,D). Moreover, some previous studies have also confirmed that the beta diversity and abundance of fungal communities are more different than those of bacterial communities with the change of environment (Liu J. L. et al., 2022; Xu et al., 2022).

Beneficial microbes play crucial roles in plant disease resistance and ecological function (Amoo and Babalola, 2017; Aziz et al., 2021). For example, previous studies have found that Trichoderma harzianum can alleviate the effects of NaCl stress (Ahmad et al., 2015) and rhizobacteria Pseudomonas sp. can effectively alleviate drought stress (Yasmin et al., 2022). Arbuscular mycorrhizal fungi (AMF) could provide host plants the essential mineral elements, and facilitate plant health (Bag et al., 2022). Other studies have found that Nitrososphaeraceae bacteria could play crucial roles in ecological function (Shen et al., 2012; Amoo and Babalola, 2017). In our research, we found that there was a significant positive correlation between Nitrososphaeraceae and ammonia oxidation and nitrification (Figure 6A), and the abundance of Nitrososphaeraceae in the two locations was different. Moreover, the abundance of Nitrososphaeraceae in Hangzhou was significantly lower than that in Shengzhou, and thus we speculate that the abundance of Nitrososphaeraceae is affected by the locations of tea garden (*p* < 0.01) (Figure 6B). Huang et al. (2021) found that AMF in the rhizosphere establish symbionts with host plants and participate in the process of plant resistance to root rot (Huang et al., 2021) and Begum et al. (2019) revealed that AMF are associated with improved resistant to drought tolerance of maize. Likewise, Bag et al. (2022) reviewed that AMF are beneficial to the growth and development of tea plants. In our study, LEfSe analysis showed that the abundance of AMF in rhizosphere fungi of Zhongcha108 was higher than that of Longjing43, and the results were the same in tea gardens of different locations (Figure 5B). We also analyzed the abundance of AMF in ITS amplicon sequencing results and verified the results of LEfSe analysis. In addition, we found that pathogenic fungi Glomerella and Colletotrichum may be related to AMF (Figure 5C). It was also found that there were significant differences in the abundance and activity of rhizosphere colonization of AMF among different mulberry varieties (Tu et al., 2021). Previous studies have also found that arbuscular mycorrhizae can colonize the rhizosphere of plants through hyphae, and help the growth and development of plants and resist the invasion of pathogenic bacteria through nutrient absorption, metabolite transfer and mineral element exchange (Li M. H. et al., 2021), and there are differences in rhizosphere microorganisms of different wheat varieties resistant to fusarium (Li et al., 2020). Although Zhongcha108 was developed through radiation treatment from Longjing43, field experiments show that Zhongcha108 can resist biotic and abiotic stress better than Longjing43 (Wang et al., 2018). However, whether the rhizosphere microorganisms could have beneficial impact on Zhongcha108 for its resistance to anthracnose has never been reported before. Therefore, we used a confocal microscope to observe Zhongcha108 and Longjing43 inoculated with arbuscular fungi, and found significant differences in the colonization of arbuscular fungi in their roots (*p* = 0.05) (Figures 7A,B). Based on the results, we boldly speculated that AMF might help Zhongcha108 to have stronger disease resistance than Longjing43, which positively answered the third question that the AMF are related to the disease resistance of Zhongcha108 as a group of potential beneficial microorganisms. Nevertheless, we acknowledge that our findings may mask the influence of other factors on fungal communities, but this study is the first to demonstrate that the resistance or suseptibitlity of tea varieties may be closely associated with the colonization of beneficial fungi in the rhizosphere.

## Conclusion

There were significant differences in soil physical and chemical properties and rhizosphere microbial communities between the two tea locations, and there were also significant differences in rhizosphere microorganisms between the two tea varieties in the same location. The analysis of PCoA and Co-occurrence confirmed that the influence of different locations on tea rhizosphere microorganisms is greater than that of different tea varieties, and fungi are more sensitive to the change in microenvironments. LDA Effect Size analysis of fungi showed that the abundance of AMF was higher in Zhongcha108 than that in Longjing43, and the difference in colonization rate of AMF between the two varieties was verified by field experiments. The rhizosphere microbial community could be influenced by both locations and varieties, however, the location is a more impactful driving factor, suggesting that rhizosphere fungi are more sensitive to changes in microenvironments. Among various microbial communities, Nitrososphaeraceae are related to the ecological function of tea gardens and AMF are related to the disease resistance of tea plants. Thus, the use of beneficial microbial communities is of great significance for the improvement of the ecological environment of tea gardens and the disease resistance of tea plants.

## Data availability statement

The data presented in the study are deposited in the BioProject database, accession number PRJNA872891. http://www.ncbi.nlm.nih.gov/bioproject/872891.

## Author contributions

XL, L-CF, and W-YH conceived and designed the research. ZZ, S-BG, QH, GJA, PY, L-PZ, Z-ZL, J-YF, and J-YZ performed the experiments and analyzed the data. XL, J-YF, W-YH, ZZ, and L-CF discussed the data. ZZ, L-CF, GJA, and XL wrote the manuscript with the contributions from the other authors. All authors contributed to the article and approved the submitted version.

## Funding

This work was funded by the National Key R&D Program of China (2020YFD10007); the Opening Fund of Provincial Key Lab of Tea Refining and Innovation Key Laboratory of Sichuan Province (SCTOF202202); Hangzhou Science and Technology Development Project (2020ZDSJ0632); and the Innovation Project of the Chinese Academy of Agricultural Sciences (CAAS-ASTIP-2019-TRICAAS). L-CF thanks to the Scientific research startup fee for introduced talents of NWAFU (Z1090222024).

## Conflict of interest

The authors declare that the research was conducted in the absence of any commercial or financial relationships that could be construed as a potential conflict of interest.

## Publisher’s note

All claims expressed in this article are solely those of the authors and do not necessarily represent those of their affiliated organizations, or those of the publisher, the editors and the reviewers. Any product that may be evaluated in this article, or claim that may be made by its manufacturer, is not guaranteed or endorsed by the publisher.
