# Supplementary material for: Diversity in rhizospheric microbial communities in tea varieties at different locations and tapping potential beneficial microorganisms
Source: Front Microbiol. 2022 Nov 10;13:1027444. doi: 10.3389/fmicb.2022.1027444 (PMC9685800; doi:10.3389/fmicb.2022.1027444)
Supplement: Supplementary file 6 [file Data_Sheet_6.docx]

**Table S1**

Relative abundance of bacteria phyla in Fig.1.

| Taxonomy | HZ-LJ43 | HZ-ZC108 | SZ-LJ43 | SZ-ZC108 | F | P |
| --- | --- | --- | --- | --- | --- | --- |
| Crenarchaeota | 0.041±0.01a | 0.041±0.01a | 0.019±0.003b | 0.025±003ab | 2.81 | 0.05 |
| Acidobacteriota | 0.219±0.03a | 0.304±0.02b | 0.168±0.006a | 0.177±0.02a | 8.72 | 0 |
| Chloroflexi | 0.056±0.004a | 0.0905±0.004a | 0.186±0.01b | 0.208±0.02b | 30.65 | 0 |
| Proteobacteria | 0.383±0.04a | 0.234±0.02b | 0.299±0.02ab | 0.277±0.04b | 4.66 | 0.01 |
| unidentified | 0.002±0.001a | 0.001±0.0001b | 0.002±0.0001a | 0.002±0a | 4.55 | 0.01 |
| Actinobacteriota | 0.110±0.01a | 0.075±0.004b | 0.085±0.005ab | 0.097±0.01ab | 3.15 | 0.04 |
| Bacteroidota | 0.015±0.002a | 0.023±0.002b | 0.029±0.003c | 0.022±0.002b | 9.01 | 0 |
| Planctomycetota | 0.038±0.006a | 0.051±0.005a | 0.062±0.11a | 0.051±0.008a | 1.64 | 0.20 |
| Verrucomicrobiota | 0.029±0.004bc | 0.063±0.004a | 0.042±0.006b | 0.025±0.004c | 15.07 | 0 |
| WPS-2 | 0.0279±0.005a | 0.0087±0.001b | 0.027±0.003a | 0.025±0.004a | 7.96 | 0.001 |

Mean values ± standard error (n=8); Different letters indicate a significant difference *(p* < 0.05) based on one-way ANOVA followed by an LSD test.

**Table S2**

Relative abundance of fungi phyla in Fig.1.

| Taxonomy | HZ-LJ43 | HZ-ZC108 | SZ-LJ43 | SZ-ZC108 | F | P |
| --- | --- | --- | --- | --- | --- | --- |
| Other | 0.012±0.002a | 0.0086±0.002a | 0.012±0.003a | 0.0066±0.003a | 1.03 | 0.39 |
| Ascomycota | 0.174±0.01ab | 0.225±0.01a | 0.13±0.03bc | 0.091±0.02c | 10.24 | 0 |
| Basidiomycota | 0.243±0.05a | 0.091±0.019b | 0.106±0.02b | 0.334±0.03a | 12.3 | 0 |
| Chytridiomycota | 0 | 0.0085±0.009a | 0 | 0 | 1 | 0.41 |
| Glomeromycota | 0.0001±0.00005a | 0.0036±0.0006b | 0.0015±0.0003a | 0.0054±0.0008c | 19.19 | 0 |
| Incertae_sedis | 0.0002±0.0001ab | 0.0003±0.0002a | 0 | 0 | 2.89 | 0.05 |
| Zygomycota | 0.0005±0.0001a | 0.0011±0.0003b | 0.0003±0.0001a | 0.0002±0.00006a | 4.76 | 0.008 |
| unidentified | 0.571±0.04a | 0.66±0.02b | 0.749±0.03b | 0.562±0.03a | 8.01 | 0.001 |

Mean values ± standard error (n=8); Different letters indicate a significant difference *(p* < 0.05) based on one-way ANOVA followed by an LSD test.

**Table S3**

Topological metrics of rhizosphere bacterial community networks in Fig.2.

| 16S Co-occurrence | HZ-LJ43/ZC108 | SZ-LJ43/ZC108 | LJ43-HZ/SZ | ZC108-HZ/SZ |
| --- | --- | --- | --- | --- |
| pos.edges | 2857 | 313 | 3041 | 2190 |
| neg.edges | 2481 | 187 | 2782 | 2033 |
| vertices | 162 | 137 | 164 | 160 |
| average degree | 65 | 7 | 71 | 52 |
| connectance | 40.90% | 5.40% | 43.60% | 33.20% |

**Table S4**

Topological metrics of rhizosphere fungal community networks in Fig.2.

| ITS Co-occurance | HZ-LJ43/ZC108 | SZ-LJ43/ZC108 | LJ43-HZ/SZ | ZC108-HZ/SZ |
| --- | --- | --- | --- | --- |
| pos.edges | 522 | 67 | 1502 | 991 |
| neg.edges | 455 | 39 | 1117 | 872 |
| vertices | 83 | 39 | 106 | 93 |
| average degree | 23 | 5 | 49 | 40 |
| connectance | 28.70% | 14.30% | 47.10% | 43.50% |

**Table S5**

Alpha diversity index of bacteria and fungi in Fig.3

| Alpha diversity | HZ-LJ43 | HZ-ZC108 | SZ-LJ43 | SZ-ZC108 | F | P |
| --- | --- | --- | --- | --- | --- | --- |
| 16S-shannon | 9.99±0.04a | 10.66±0.08b | 10.69±0.07b | 10.43±0.04c | 23.854 | 0 |
| ITS-shannon | 6.77±0.11a | 6.75±0.15a | 4.97±0.20c | 5.71±0.13b | 30.741 | 0 |

Mean values ± standard error (n=8); Different letters indicate a significant difference *(p* < 0.05) based on one-way ANOVA followed by an LSD test.

**Table S6**

Reads abundance of related fungi in Fig.5

| Taxonomy | HZ-LJ43 | HZ-ZC108 | SZ-LJ43 | SZ-ZC108 | F | P |
| --- | --- | --- | --- | --- | --- | --- |
| Acaulospora | 8.25±3.12a | 292±95.81b | 26.25±10.49a | 157.125±46.58ab | 6.07 | 0.003 |
| Gigaspora | 20.875±10.38a | 116.375±23.60b | 168.25±31.85b | 204.25±43.22b | 7.16 | 0.001 |
| Glomus | 0 | 212.875±97.43ab | 85.75±38.05ab | 138.875±74.55 | 1.95 | 0.15 |
| Rhizophagus | 2±2b | 0 | 61.125±20.65b | 751.75±145.45a | 24.88 | 0 |
| Glomerella | 273.125±71.48a | 352.125±75.06ab | 573±194.01ab | 793.125±206.45b | 2.41 | 0.09 |
| Colletotrichum | 38±17.09a | 4.125±4.12b | 0 | 0 | 4.39 | 0.01 |

Mean values ± standard error (n=8); Different letters indicate a significant difference *(p* < 0.05) based on one-way ANOVA followed by an LSD test.

**Table S7**

Reads abundance of related bacteria in Fig.6

| Taxonomy | HZ-LJ43 | HZ-ZC108 | SZ-LJ43 | SZ-ZC108 | F | P |
| --- | --- | --- | --- | --- | --- | --- |
| Nitrososphaeraceae | 0.625±0.26a | 2.625±0.86a | 34.375±9.78b | 37.625±4.53b | 13.53 | 0 |
| Edaphobacter | 117.375±19.40a | 271.375±32.04b | 238.125±20.43b | 149.875±31.65a | 7.44 | 0.001 |
| Subgroup_13 | 2873.5±515.02a | 1365±109.75b | 1052.875±142.21b | 537.625±99.32b | 13.12 | 0 |
| Gaiellales | 20.25±2.08b | 47.875±3.78a | 46.875±4.35a | 58.375±9.32a | 8.48 | 0 |
| Chitinophagaceae | 8±1.13a | 31±4.30b | 37.875±5.50b | 8.625±1.83a | 17.61 | 0 |
| Thermosporothrix | 9.875±3.18a | 46.125±5.95b | 29.5±9.21b | 31.625±5.16b | 5.65 | 0.004 |
| Paenibacillus | 53.25±6.95a | 19.5±1.70c | 37±5.95b | 30.375±6.24bc | 6.35 | 0.002 |
| Ellin6067 | 10.5±1.32a | 57.875±6.33b | 63.125±14.50b | 40.5±5.42b | 7.99 | 0.001 |

Mean values ± standard error (n=8); Different letters indicate a significant difference *(p* < 0.05) based on one-way ANOVA followed by an LSD test.
